# Intracranial complications of sinogenic and otogenic infections in children: an ESPN survey on their occurrence in the pre-COVID and post-COVID era

**DOI:** 10.1007/s00381-024-06332-9

**Published:** 2024-03-08

**Authors:** L. Massimi, G. Cinalli, P. Frassanito, V. Arcangeli, C. Auer, V. Baro, A. Bartoli, F. Bianchi, S. Dietvorst, F. Di Rocco, P. Gallo, F. Giordano, J. Hinojosa, S. Iglesias, V. Jecko, G. Kahilogullari, F. Knerlich-Lukoschus, R. Laera, D. Locatelli, D. Luglietto, M. Luzi, M. Messing-Jünger, R. Mura, P. Ragazzi, L. Riffaud, J. Roth, A. Sagarribay, M. Santos Pinheiro, P. Spazzapan, P. Spennato, N. Syrmos, G. Talamonti, L. Valentini, M. L. Van Veelen, M. Zucchelli, G. Tamburrini

**Affiliations:** 1 Pediatric Neurosurgery, Neuroscience-Sense Organs-Chest Department, Fondazione Policlinico Universitario A. Gemelli IRCCS, Rome, Italy; 2 Department of Neuroscience, Catholic University Medical School, Rome, Italy; 3 Santobono-Pausilipon Children’s Hospital, AORN, Naples, Italy; 4 Clinical Psychology Unit, Fondazione Policlinico Universitario A. Gemelli IRCCS, Rome, Italy; 5 Department of Neurosurgery, Johannes Kepler University Linz, Kepler University Hospital GmbH, Linz, Austria; 6 Pediatric and Functional Neurosurgery, Department of Neurosciences, University of Padova, Padua, Italy; 7 Department of Neurosurgery, Geneva University Hospitals, Geneva, Switzerland; 8 University Hospitals Leuven, Leuven, Belgium; 9 Hôpital Femme-Mère-Enfant, Université de Lyon, Lyon, France; 10 Birmingham Children’s Hospital, Birmingham, UK; 11 University of Florence, Florence, Italy; 12 Hospital Sant Joan de Déu, Barcelona, Spain; 13 Hospital Regional Universitario de Malaga, Malaga, Spain; 14 Centre Hospitalier Universitaire de Bordeaux, Bordeaux, France; 15 Department of Neurosurgery, Ankara University, Ankara, Turkey; 16 Division Pediatric Neurosurgery, Department of Neurosurgery, University Medical Center Göttingen, Göttingen, Germany; 17 Neurosurgery Department, Università Dell’Insubria, Ospedale di Circolo e Macchi Foundation, Varese, Italy; 18 Ospedale Pediatrico Bambino Gesù, Rome, Italy; 19 Azienda Ospedaliero Universitaria Delle Marche, Ancona, Italy; 20 Asklepios Children’s Hospital, St. Augustin, Germany; 21 Meyer Children’s Hospital IRCCS, Florence, Italy; 22 Department of Pediatric Neurosurgery, Ospedale Infantile Regina Margherita, Città della Salute e della Scienza, Turin, Italy; 23 Rennes University Hospital, Rennes, France; 24 Dana Children’s Hospital, Tel Aviv Medical Center, Tel Aviv University, Tel Aviv, Israel; 25 Hospital Dona Estefânia-Centro Hospitalar Universitário, Lisboa, Portugal; 26 Centro Hospitalar Lisboa Norte-Hospital Santa Maria, Lisboa, Portugal; 27 University Medical Center-Ljubljana, Ljubljana, Slovenia; 28 Aristotle University of Thessaloniki, Thessaloniki, Greece; 29 ASST Niguarda, Milan, Italy; 30 Fondazione IRCCS Istituto Neurologico Carlo Besta, Milan, Italy; 31 Erasmus MC Sophia Children’s Hospital, Rotterdam, Netherlands; 32 IRCCS Azienda Ospedaliero-Universitaria di Bologna, Istituto Scienze Neurologiche Di Bologna, Boulogne, Italy; 33 Hospital CUF Descobertas, Lisboa, Portugal

**Keywords:** Intracranial empyema, Brain abscess, Sinusitis, Otitis, COVID, Pediatric neurosurgery

## Abstract

**Background:**

COVID-19 pandemic is thought to have changed the epidemiology of some pediatric neurosurgical disease: among them are the intracranial complications of sinusitis and otitis (ICSO). According to some studies on a limited number of cases, both streptococci-related sinusitis and ICSO would have increased immediately after the pandemic, although the reason is not clear yet (seasonal changes versus pandemic-related effects). The goal of the present survey of the European Society for Pediatric Neurosurgery (ESPN) was to collect a large number of cases from different European countries encompassing the pre-COVID (2017–2019), COVID (2020–2021), and post-COVID period (2022–June 2023) looking for possible epidemiological and/or clinical changes.

**Material and methods:**

An English language questionnaire was sent to ESPN members about year of the event, patient’s age and gender, presence of immune-deficit or other favoring risk factors, COVID infection, signs and symptoms at onset, site of primary infection, type of intracranial complication, identified germ, type and number of surgical operations, type and duration of medical treatment, clinical and radiological outcome, duration of the follow-up.

**Results:**

Two hundred fifty-four cases were collected by 30 centers coming from 14 different European countries. There was a statistically significant difference between the post-COVID period (129 children, 86 cases/year, 50.7% of the whole series) and the COVID (40 children, 20 cases/year, 15.7%) or the pre-COVID period (85 children, 28.3 cases/year, 33.5%). Other significant differences concerned the presence of predisposing factors/concurrent diseases (higher in the pre-COVID period) and previous COVID infection (higher in the post-COVID period). No relevant differences occurred as far as demographic, microbiological, clinical, radiological, outcome, morbidity, and mortality data were concerned. Paranasal sinuses and middle ear/mastoid were the most involved primary site of infection (71% and 27%, respectively), while extradural or subdural empyema and brain abscess were the most common ICSO (73% and 17%, respectively). Surgery was required in 95% of cases (neurosurgical and ENT procedure in 71% and 62% of cases, respectively) while antibiotics in 99% of cases. After a 12.4-month follow-up, a full clinical and radiological recovery was obtained in 85% and 84% of cases, respectively. The mortality rate was 2.7%.

**Conclusions:**

These results suggest that the occurrence of ICSO was significantly increased after the pandemic. Such an increase seems to be related to the indirect effects of the pandemic (e.g., immunity debt) rather than to a direct effect of COVID infection or to seasonal fluctuations. ICSO remain challenging diseases but the pandemic did not affect the management strategies nor their prognosis. The epidemiological change of sinusitis/otitis and ICSO should alert about the appropriate follow-up of children with sinusitis/otitis.

**Supplementary Information:**

The online version contains supplementary material available at 10.1007/s00381-024-06332-9.

## Introduction

COVID-19 pandemic has had an impact in the neurosurgical clinical practice either in changing the way to take care of patients [1, 2] or changing the risk of some disease like tumors or brain injuries [3–6]. During recent meetings of the European Society for Pediatric Neurosurgery (ESPN), a certain feeling about a possible change also in the epidemiology of intracranial complications of sinusitis and otitis (ICSO) started circulating based on personal anecdotal reports. In 2022, the Centers for Disease Control and Prevention (CDC) provided a study to investigate reports on a possible increase of these intracranial complications in the USA [7, 8], thus suggesting that the same “feeling” was shared also outside Europe [9]. The CDC analysis actually showed an increase of streptococci-related intracranial empyema or abscess during the 2021–2022 period but this trend was considered as part of seasonal fluctuations, as demonstrated by the decline during the second half of 2022. Moreover, the peak did not correlate with an increased number of admissions in intensive care units or an increase of the mortality rate.

In 2022, another survey on this topic was launched in the USA because of the report of a 236% increase of infectious intracranial complications in a single hospital [10]. In this instance, the Emergency Infections Network (EIN) recruited 8 USA centers and investigated the trend of streptococcus-related sinusitis and oto-mastoiditis during the pre-COVID (January 2018–January 2020) and the COVID period (March 2020–March 2022) in children. The results showed a relevant increase of intracranial infections (100.9%) and sinusitis complicated by intracranial infections (76.7%) together with a decrease of orbital cellulitis (14.5%), sinusitis (31.9%), mastoiditis (24.7%), and mastoiditis complicated by intracranial infection (116.7%). The partial discrepancy among this data is explained by some biases like the few number of participating centers, the voluntary participation to the study, the limited data collected, and the absence of details on patients (number of patients and demographic findings were not provided). However, the value of this survey was to raise the problem and to point the need of further studies.

The goal of the present survey is actually to provide further information on this issue by expanding the number of centers and the number of patients other than by prolonging the investigating period to the post-COVID time.

## Materials and methods

The survey was addressed to all ESPN members. It was advertised by mail and through the Society’s website other than during ESPN meetings and courses, starting from the beginning of 2023. The goal was to analyze the impact of ICSO in the neurosurgical practice during the COVID and post-COVID era and to compare that with the pre-COVID era. For that reason, only patients requiring or candidate to surgery were considered. The period ranging from January 2020 to December 2021 was considered as COVID era, because of the occurrence of the lockdown period and the further waves of the pandemic. The period going from January 2022 to June 2023 (deadline of the survey) was considered as post-COVID era, due to the drop of COVID infection with gradual coming back to the normality both in the daily life and in the clinical practice. Finally, the time period ranging from January 2017 to December 2019 was used to collect data on the pre-COVID era. Exclusion criteria were represented by absence of sinusitis or otitis/mastoiditis, presence of postoperative or surgical site or CSF shunt-related infections, primary meningitis.

An English language questionnaire on Google Forms defining the following items was created: year of the event, patient’s age and gender, presence of immune-deficit or other favoring risk factors, COVID infection, signs and symptoms at onset, site of primary infection, type of intracranial complication, identified germ, type and number of surgical operations, type and duration of medical treatment, clinical and radiological outcome, duration of the follow-up (see Supplementary file).

Statistical analysis was carried out through the use of data analysis software “IBM SPSS Statistics 29” (Statistical Package for Social Science). For the sample identification, descriptive statistics and frequency analyses of demographic variables were realized. For the detection of the statistically significant differences among the different periods of the survey, Analysis of Variance (ANOVA) was used. Correlations with Pearson’s *r* were performed to highlight the presence of significant correlations. The significance of *p* value was set at < 0.05.

## Results

### Demographics

**Fig. 1:**
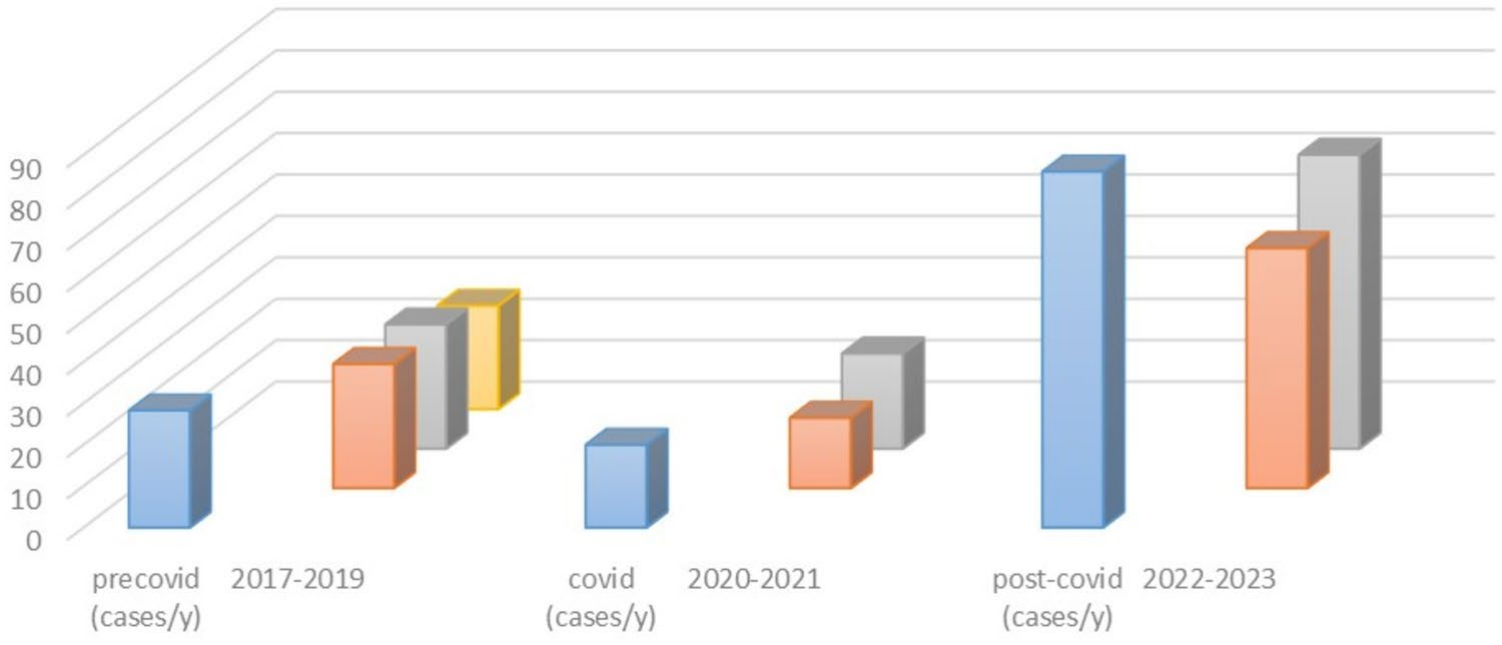
Diagram showing the trend of the cases in the period of study. Light blue columns show the prevalence of intracranial complications of sinusitis/otitis in the pre-COVID, COVID, and post-COVID era. Colored (orange, gray, and yellow) columns show the number of cases per year in each era

Overall, 254 cases from 31 European centers were collected, 14 different European countries being represented (Table 1). Eighty-five cases (33.5%) belonged to the pre-COVID era, 30 of them being treated in 2017 (11.8%), 30 in 2018 (11.8%), and 25 in 2019 (9.8%). The remaining 169 cases (66.5%) were admitted during the COVID and post-COVID era. Namely, 17 cases were observed in 2020 (6.6%) and 23 in 2021 (9%), that is during the COVID era (40 cases, 15.7%), while 58 in 2022 (23%) and 71 in 2023 (28%), that is in the post-COVID era (129 cases, 50.8%). Figure 1 summarizes the trend of cases over the years. Overall, the mean age of recruited patients was 10.17 years (range 1–18 years), with a 1.9 male/female ratio (167 boys and 87 girls). The difference in number of cases, age, and sex, according to the different periods, is reported on Table 2.

**Table 1:** Participating centers and case distribution over the time

Participating centers	*N* of cases	Pre-COVID era (2017–2019)	COVID era (2020–2021)	Post-COVID era (2022–2023)
Birmingham Children’s Hospital-Birmingham (UK)	26	11	4	11
Hôpital Femme-Mère-Enfant-Lyon (France)	24	13	3	8
Santobono-Pausilipon Hospital-Naples (Italy)	22	7	0	15
Rennes University Hospital-Rennes (France)	17	4	6	7
Hospital Regional Universitario-Malaga (Spain)	14	6	2	6
Ospedale Pediatrico Bambino Gesù-Rome (Italy)	14	2	3	9
Pediatric Neurosurgery, University Hospital-Bordeaux (France)	13	6	0	7
IRCCS Istituto Scienze Neurologiche di Bologna-Boulogne (Italy)	13	6	0	7
Asklepios Children´s Hospital-St. Augustin (Germany)	13	4	1	8
Hospital Sant Joan De Déu-Barcelona (Spain)	11	0	3	8
University Hospitals-Leuven (Belgium)	11	6	1	4
Meyer Children’s Hospital IRCCS-Florence (Italy)	11	3	3	5
University of Florence/Meyer Children’s Hospital IRCCS-Florence (Italy)	11	5	2	4
Fondazione Policlinico Universitario A. Gemelli IRCCS-Rome (Italy)	7	1	1	5
ASST Sette Laghi-Varese (Italy)	6	4	1	1
Hospital Dona Estefânia-Centro Hospitalar Universitário-Lisboa (Portugal)	6	1	3	2
Hôpitaux Universitaires de Genève-Geneva (Switzerland)	5	0	0	5
Dana Children’s Hospital-Tel Aviv (Israel)	4	1	2	1
Johannes Kepler University Hospital-Linz (Austria)	4	1	1	2
Ospedale Infantile Regina Margherita-Turin (Italy)	3	1	0	2
Department of Neuroscience, University of Padova-Padua (Italy)	3	2	0	1
University Medical Center-Ljubljana (Slovenia)	3	0	1	2
Pediatric Neurosurgery, University Medical Center-Göttingen (Germany)	3	0	0	3
ASST Niguarda-Milan (Italy)	2	1	0	1
Aristotle University of Thessaloniki-Thessaloniki (Greece)	2	0	1	1
Azienda Ospedaliero Universitaria delle Marche-Ancona (Italy)	1	0	0	1
Centro Hospitalar Lisboa Norte-Hospital Santa Maria-Lisboa (Portugal)	1	0	0	1
Fondazione IRCCS Istituto Neurologico Carlo Besta-Milan (Italy)	1	0	1	0
Hospital CUF Descobertas-Lisboa (Portugal)	1	0	0	1
Ankara University Department of Neurosurgery-Ankara (Turkey)	1	0	1	0
Erasmus MC Sophia Children’s Hospital-Rotterdam (Netherlands)	1	0	0	1
Overall	254	***N of cases/year***		
		***28.3***	***20***	***86***

**Table 2:** Main demographic findings

	No. of cases	Mean age (years)	M/F ratio (boys/girls)	No. of cases*	Mean age* (years)	M/F ratio* (boys/girls)
2017	30	11.3	1.1 (16/14)	85	10.37	1.6 (53/32)
2018	30	10.8	2.7 (22/8)			
2019	25	8.6	1.5 (15/10)			
2020	17	10.7	1.8 (11/6)	40	10.9	1.8 (26/14)
2021	23	11.8	1.8 (15/8)			
2022	58	10.4	2.4 (41/17)	129	9.8	2.14 (88/41)
2023	71	9.3	1.9 (47/24)			
Overall	254	10.17	1.9 (167/87)	254	10.17	1.9

^*^Pre-COVID (2017–2019), COVID (2020–2021), and post-COVID era (2022–2023)

The difference in number of cases between pre-COVID period (2017–2019) and COVID/post-COVID period (2020–2023) was significant (85 vs 169 cases, *p* < 0.001). Similarly, the different annual incidence among the 3 periods was relevant (28.3 versus 20 versus 86 cases/year, *p* < 0.001). As far as the age of patients is concerned, the average showed similar figures over the years and across the 3 periods. The trend towards a lower age observed in 2023 was not statistically significant. A predominance of the male sex was noticed. However, the difference between boys and girls in the whole series as well as the increase of the M/F ratio through the 3 periods was not statistically relevant.

### Clinical findings

A risk factor for sinusitis/otitis and its complication was reported in a minority of cases. Eleven out of 254 children (4.3%) were immunocompromised. On the other hand, 23 patients (9%) showed other potential predisposing factors or coincidental medical conditions, most of them related to airways or ear inflammatory diseases (Table 3). The incidence of risk factors was significantly higher in the pre-COVID era compared with the other two periods (*p* = 0.001).

**Table 3:** Possible risk factors/concurrent medical conditions

Year	Immunodeficit	Other risk factors*	Previous COVID infection	Concurrent
2017	3	4	/	/
2018	1	5	/	/
2019	None	None	/	/
2020	None	2	1	None
2021	None	4	3	3
2022	1	5	15	None
2023	6	3	14	None
Overall	11	23	33	3

^*^Recurrent sinusitis (2 cases), recurrent otitis (1), chronic pharyngitis (1), bronchitis (1), asthma (1), mononucleosis (1), pediatric inflammatory multisystemic syndrome (1), orbital cellulitis (1), recurrent acute bronchiolitis and middle otitis (1), severe cochleo-vestibular malformation (1), vancomycin-induced DRESS syndrome (1), meningitis (1), Crohn’s disease (1) Moebius syndrome + cognitive delay + genetic Xp deletion + tracheomalacia (1), obesity (2), visceral adiposity (1), fibrous dysplasia (1), trisomy 21 (1), traumatic brain injury and cranioplasty (1), Chiari I malformation (1), autism (2)

A previous and a concurrent COVID infection was detected in 33 (19.5%) and 3 cases (1.7%) out of 169 patients of the COVID/post-COVID era, respectively. The great majority of children with detected previous COVID infection belonged to the 2022 (15 cases) or the 2023 period (14 cases), while only 4 cases belonged to the 2020 (1 case) or the 2021 period (3 cases). These differences between COVID and post-COVID era were highly significant (*p* < 0.001). All 3 patients with concurrent COVID infection were part of the 2021 period (Table 3).

The main presenting symptom was fever, being detected in 201 cases of the whole series (79%). Overall, other symptoms occurred in 214 patients (84%) (Table 4). These symptoms were represented by neurological symptoms and signs, including hemiparesis, cranial nerve palsy, aphasia, ataxia, cerebellar syndrome, fatigue, and headache (see Table 4 for details). Finally, a pure radiological diagnosis (no clinical symptoms) was done in 7 cases (2.7%), equally distributed over the time.

**Table 4:** Presenting symptoms, primary site of infection, and primary type of complication

Year	Fever	Other symptoms*	Paranasal sinus	Other location	Extradural empyema	Subdural empyema	Brain abscess	Multiple locations^	Venous sinus thrombosis
2017	22 (73%)	24 (80%)	24 (80%)	6 (20%)	8 (27%)	11 (37%)	8 (27%)	4 (13%)	2 (7%)
2018	24 (80%)	27 (90%)	23 (77%)	7 (23%)	12 (40%)	12 (40%)	5 (17%)	5 (17%)	6 (20%)
2019	19 (76%)	19 (76%)	14 (56%)	11 (44%)	9 (36%)	10 (40%)	4 (16%)	5 (20%)	6 (24%)
2020	13 (76%)	17 (100%)	14 (82%)	3 (18%)	7 (41%)	6 (35%)	3 (17%)	4 (23%)	2 (11%)
2021	16 (70%)	20 (87%)	17 (74%)	6 (26%)	8 (35%)	8 (25%)	4 (17%)	3 (13%)	1 (4%)
2022	53 (91%)	51 (88%)	47 (81%)	11 (19%)	23 (40%)	26 (45%)	9 (15%)	21 (36%)	9 (15%)
2023	54 (76%)	56 (79%)	42 (59%)	29 (41%)	30 (42%)	23 (32%)	13 (18%)	15 (21%)	18 (25%)
Overall	201 (79%)	214 (84%)	181 (71%)	73 (29%)	97 (38%)	96 (37%)	42 (17%)	57 (22%)	44 (17%)

^*^Hemiparesis, cranial nerve palsy, aphasia, ataxia, cerebellar syndrome, fatigue, and confusion in 80 cases (31%), headache in 107 cases (42%), frontal/orbital/retro-auricular swelling or signs of orbital cellulitis in 41 cases (16%), seizures and vomiting in 38 cases each (15%), drowsiness/lethargy/coma in 33 cases (12%), ear pain in 15 cases (6%), nasal obstruction/rhinorrhea in 8 cases (3%), meningitis signs/symptoms in 7 cases (2.7%), anorexia in 4 cases (1.5%), otorrhea and diarrhea in 3 cases each (1.1%), hydrocephalus in one case (0.5%)

^Extradural ± subdural ± brain abscess

Paranasal sinuses were largely the most involved primary site of infection (181 cases, 71%), followed by middle ear/mastoid (68 cases, 27%), orbit (4 cases, 1.5%), and dental space (1 case, 0.5%) (Table 4). Primary intracranial complications were present at the diagnosis and represented mainly by extradural or subdural empyema (186 cases, 73%) and brain abscess (42 cases, 17%) (Table 4). Cerebritis (15 cases, 6%), dural impregnation (6 cases, 2%), intraorbital abscess (3 cases, 1.1%), and infected subdural hygroma (2 cases, 0.9%) were the other primary complications. Secondary complications (venous sinus thrombosis, meningoencephalitis, dural impregnation, hydrocephalus, ventriculitis, cerebritis, brain abscess, cavernous sinus abscess, brain edema, brain infarction, orbital or dental abscess, cerebellar tonsil herniation, septic thrombosis, osteomyelitis) further complicated the initial presentation and were reported in 112 cases (44%), with similar distribution over the time (6 cases in 2017, 8 in 2018, 12 in 2019, 8 in 2020, 9 in 2021, 28 in 2022, and 41 in 2023). No statistically relevant difference in the distribution of symptoms, primary site of infection, and type of complication was detected among the 3 periods or the different years.

A responsible germ was identified in 176 cases (69%), while a negative culture was found in the remaining 78 cases (31%). The detected bacteria are reported in detail on Table 5. All but two patients underwent a medical treatment consisting of antibiotics for a mean duration of 5.8 weeks. Overall, 14% of children received a > 10-week-long antibiotic treatment. Steroids or other anti-inflammatory drugs were simultaneously administered in 56% of cases of the whole series. No significant differences among the 3 periods and the different years were detected about the occurrence of positive cultures or therapeutic strategies (Table 5).

**Table 5:** Identified germs and medical treatment

Year	Positive cultures	Identified germs	Antibiotic treatment	Mean duration of treatment	Other medical treatment*
2017	17 (56%)	*S. anginosus* (6), *S. intermedius* (4), *S. pneumoniae* (2), *S. pyogenes* (1), Strept. group F (1), *F. necrophorum* (2), *S. epidermidis* (1), *S. aureus* (1)	29 (97%)	> 10 weeks in 8 cases (27%) Mean duration in the remaining cases: 5.6 weeks	16 (53%)
2018	21 (70%)	*S. intermedius* (5), *S. anginosus* (3), *S. pyogenes* (1), *S. milleri* (2), *S. pneumoniae* (1), *S. epidermidis* (4), *S. constellatus* (1), *H. influenzae* (1), *H. haemolyticus* (1), *P. aeruginosa* (1), *N. macacae* (1), *P. loescheii* (1), *F. necrophorum* (2), *F. nucleatum* (1)	30 (100%)	6.4 weeks	13 (43%)
2019	18 (72%)	*S. intermedius* (3), *S. anginosus* (1), *S. pyogenes* (4), *P. anaerobius* (2), *S. epidermidis* (1), *S. constellatus* (1), *S. aureus* (1), *P. aeruginosa* (1), *F. necrophorum* (3), *E. faecalis* (1), *P. acnes* (1), *Bacteroides* spp. (2), *Actinomyces* spp. (1)	24 (96%)	> 10 weeks in 3 cases (12%) Mean duration in the remaining cases: 4.9 weeks	12 (48%)
2020	15 (88%)	*S. intermedius* (7), *S. constellatus* (1), *H. influenzae* (2), *S. aureus* (2), *S. epidermidis* (2), *F. nucleatum* (1), *E. corrodens* (1), *M. tuberculosis* (1), *C. albicans* (1)	17 (100%)	> 10 weeks in 3 cases (17%) Mean duration in the remaining cases: 6.7 weeks	11 (64%)
2021	16 (70%)	*S. intermedius* (5), *S. constellatus* (3), *S. pneumoniae* (3), *S. anginosus* (1), Strept. group F (1), *H. influenzae* (1), *S. aureus* (1), *P. micra* (1), *E. cloacae* (1), *A. xylosoxidans* (1)	23 (100%)	> 10 weeks in 6 cases (26%) Mean duration in the remaining cases: 6.0 weeks	13 (56%)
2022	42 (72%)	*S. intermedius* (17), *S. pneumoniae* (4), *S. anginosus* (7), *S. constellatus* (4), *S. milleri* (1), *P. anaerobius* (1), *S. mitis* (1), *M. catarrhalis* (1), *S. aureus* (3), *H. influenzae* (2), *F. necrophorum* (2), *F. nucleatum* (1), *S. exiquia* (1), *S. moorei* (1), *D. pneumosintes* (1), *E. corrodens* (4), *C. albicans* (1), *P. micra* (1), *P. oralis* (1), *P. loescheii* (1), *A. europeus* (1)	58 (100%)	> 10 weeks in 12 cases (20%) Mean duration in the remaining cases: 6.1 weeks	35 (60%)
2023	47 (66%)	*S. intermedius* (16), *S. anginosus* (8), *S. tonsillaris* (1), *S. pneumoniae* (3), *S. pyogenes* (8), *S. mitis* (1), Strept. group F (2), *S. constellatus* (1), *C, singulare* (1), *S. lugdunensis* (1), *S. aureus* 3), *S. epidermidis* (1), *F. necrophorum* (1), *P. oralis* (1), *P. loescheii* (1), Coccobacilli ssp. (1), *Veillonella* spp. (1), *E. coli* (1), *H. influenzae* (1), *A. niger* (1), *A. fumigatus* (1)	71 (100%	> 10 weeks in 5 cases (7%) Mean duration in the remaining cases: 5.5 weeks	42 (59%)
Overall	176 (69%)	/	252 (99%)	> 10 weeks: 37 cases (14%) Mean duration: 5.8 weeks	142 (56%)

^*^Steroids and/or anti-inflammatory drugs

The large majority of patients (242 cases, 95%) required a surgical evacuation of the intracranial infected collection, only 12 patients being managed by medical treatment alone (Table 6). The surgical evacuation was realized mainly by craniotomy (48%) or burr holes (23%) while ENT toilette of the infected cavity was carried out in 62% of cases. Additional surgery was required in 26% of cases (see Table 6 for details). A single surgical operation was enough in 142 patients (56%), while 32 of them needed 3 or more operations (13%). All these differences did not result statistically relevant, including the lower rate of multiple operations in 2023 (5.6%).

**Table 6:** Surgical management

Year	Surgery	Burr hole	Craniotomy	ENT toilette	Other*	Single operation	≥ 3 operations
2017	29 (97%)	4 (13%)	18 (60%)	19 (63%)	9 (30%)	17 (56%)	5 (16%)
2018	28 (93%)	9 (30%)	13 (43%)	19 (63%)	7 (23%)	15 (50%)	4 (13%)
2019	22 (88%)	3 (12%)	13 (52%)	17 (68%)	4 (16%)	13 (52%)	4 (16%)
2020	17 (100%)	4 (23%)	9 (53%)	9 (53%)	6 (35%)	9 (53%)	3 (17%)
2021	22 (95%)	4 (17%)	14 (61%)	14 (61%)	4 (17%)	10 (43%)	3 (13%)
2022	56 (96%)	16 (57%)	29 (50%)	38 (65%)	13 (22%)	33 (57%)	9 (15%)
2023	68 (95%)	18 (25%)	26 (36%)	42 (59%)	22 (31%)	45 (63%)	4 (5.6%)
Overall	242 (95%)	58 (23%)	122 (48%)	158 (62%)	66 (26%)	142 (56%)	32 (13%)

^*^External ventricular or subdural drainage, neuroendoscopic lavage, decompressive craniectomy, mastoidectomy, anterior ethmoidectomy, meatotomy, myringotomy, eyelid incision, VP shunt, ICP invasive monitoring

**Fig. 2:**
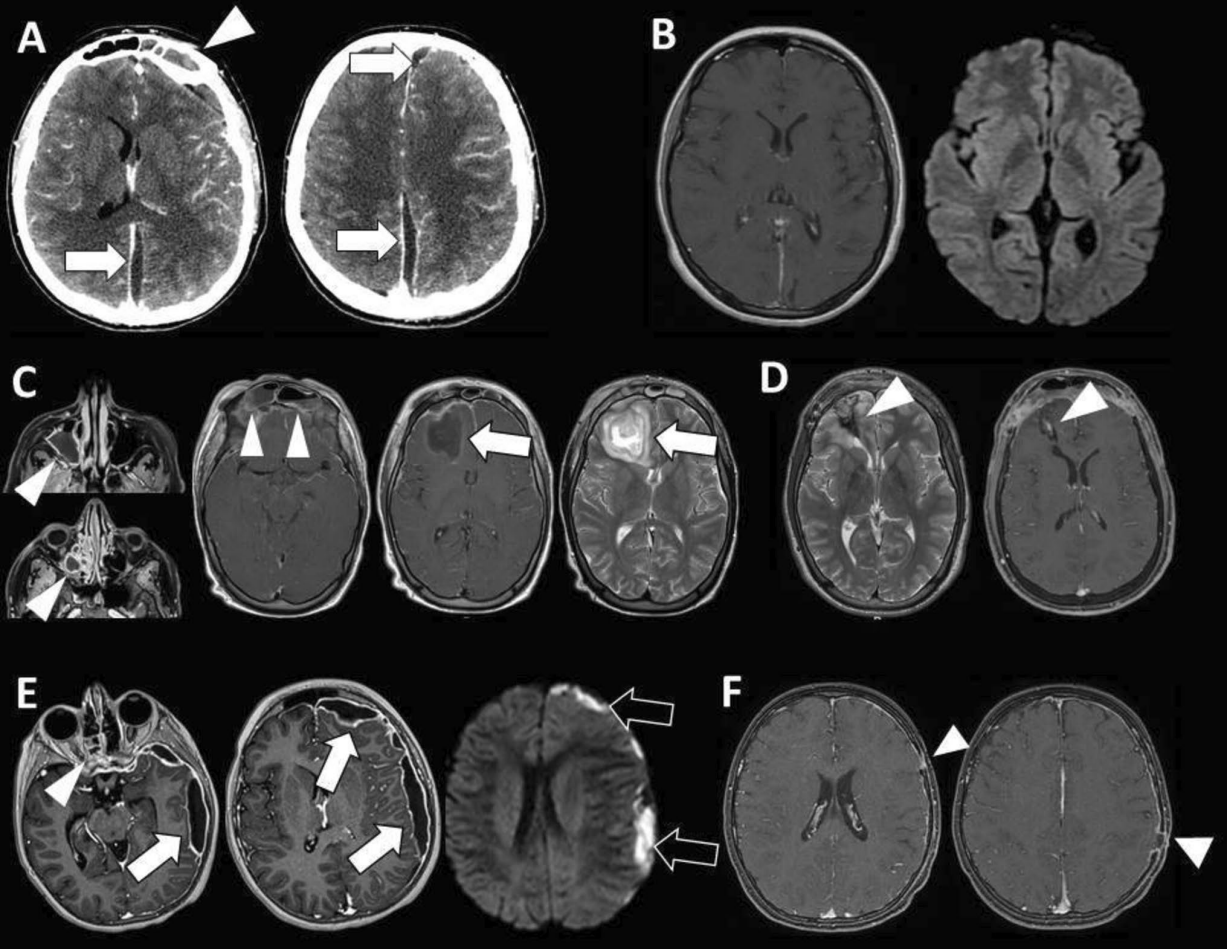
Case #1: left interhemispheric subdural empyema (**A**, arrows) complicating frontal sinusitis (**A**, arrowhead). The empyema was drained by two paramedian burr holes and the sinusitis by ENT toilette. *S. intermedius* was identified as responsible germ. Systemic antibiotic therapy cured the infection as confirmed by follow-up MR (**B**). Case #2: frontal abscess (**C**, arrow) complicating extensive sinusitis of the maxillary sinus, ethmoid cells, and frontal sinus (**C**, arrowheads). The lesion was surgical excised through bifrontal craniotomy with cranialization of the frontal sinus. *Aspergillus fumigatus* and *Aspergillus niger* were identified as responsible germs. Antimycotic treatment cured the infection with residual gliosis and minimal enhancement on follow-up MR (**D**, arrowheads). Case #3: sinusitis of the ethmoid cells (**E**, arrowhead) with left hemispheric subdural empyema (**E**, arrows) showing restriction on diffusion weighted image (**E**, black arrows). The empyema was drained by two burr holes and cured with wide spectrum systemic antibiotic therapy. Follow-up MR show the signs of the previous burr holes (**F**, arrowheads) with minimal residual dural enhancement on the left side

These treatments accounted for a favorable outcome (complete clinical recovery) in 216 cases (85%), with a complete radiological resolution recovery in 214 cases (84%) (Fig. 2). Permanent deficits and radiological remnants were equally distributed over the time (see Table 7 for details). Only two cases of recurrent subdural empyema were detected (both in 2023).

**Table 7:** Outcome*

Year	Complete clinical recovery	Residual deficits	Complete radiological recovery	Residual radiological findings	Deaths	Mean follow-up (months)
2017	24 (80%)	Tetraspasticity (1), hearing loss (1), seizures (1), memory and attention deficit (1), hemiparesis (1)	27 (90%)	White matter lesions (2), brain edema (1)	2 (6.5%)	21.4
2018	25 (83%)	Seizure (3), hemiparesis (1), spasticity (1), ataxia (1), aphasia (1), hearing loss (1), memory deficit (1), peripheral neuropathy (1)	29 (97%)	Gliosis and persistent meningeal impregnation (1)	None	23.7
2019	22 (88%)	Hemiparesis (1), speech disorders (1), hearing loss (1)	24 (96%)	Gliosis (1)	None	24.3
2020	15 (88%)	Hemiparesis (1), tetraparesis (1)	17 (100%)	None	None	16.1
2021	18 (78%)	Hemiparesis (1), seizures (2), hemianopsia (1), aphasia (1), dehydration (1)	19 (82%)	Cerebritis (1), meningeal impregnation (1), ischemia (1), bone gap (1)	1 (4%)	12.5
2022	53 (91%)	Facial paresis (1), peripheral neuropathy (1), sequelae of infected of cranioplasty (1)	52 (89%)	Meningeal impregnation (4), residual empyema (2)	2 (3.5%)	7.2
2023	59 (83%)	Hemiparesis (3), raised ICP signs (1), cranial nerve palsy (1), behavioral problems (1), speech problems (1)	46 (64%)	Meningeal impregnation (3), residual empyema (8), sinus thrombosis (2)	2 (2.8%)	2.3
Overall	216 (85%)	/	214 (84%)	/	7 (2.7%)	12.4

^*^Still ongoing treatment in 10 cases (all belonging to the 2023 period)

The mean follow-up is 12.4 months (range, 0.5–77 months). All but 7 patients (mortality rate, 2.7%) are alive at current follow-up (no statistical differences). The causes of death were complications of meningitis (3 cases), massive brain edema (2), central dysregulation and apallic syndrome (1), and acute hepatic failure (1). In 11 children (4.3%), the medical treatment is still ongoing.

## Discussion

### Background

Many studies have been carried out on the impact of COVID-19 pandemic on the pediatric population and, among those concerning the topic of the present survey, several of them are focused on the changing epidemiology and care of respiratory infection and sinusitis/otitis [11–13]. Generally, a relevant proportion of respiratory tract infections is complicated by sinusitis (5–10%), which usually show a benign course in children [14, 15]. The intracranial complications of sinusitis or otitis (extradural or subdural empyema, cerebral abscess), although not frequent, are well known in clinical practice, being described in numerous series in the literature [16–18]. On the other hand, the correlation between COVID-19-related respiratory infection/sinusitis and ICSO is a new matter of study, being sporadically reported so far [19, 20]. To date, apart from the aforementioned studies from CDC [9] and EIN [10], three articles have addressed, more or less directly, the issue of the relationship between COVID-19 pandemic and ICSO [21–23]. In spite of the low number of cases collected by each study, they represent a valuable background for the present survey.

In their study focused on the “concordance between endoscopic sinus sampling and intracranial sampling for microbiological diagnosis of intracranial infection,” Kameda-Smith and coworkers reported on 31 children collected in the 2019–2022 period (males 55%, mean age 9.92 years) presenting with subdural empyema (61%) and/or extradural empyema (29%) and/or brain abscess (19%) [23]. These patients were surgically managed by craniotomy (61%) or burr hole evacuation of the suppurative complication (39%), 48% of them needing an ENT procedure too. The authors’ conclusion (based also on a review of the literature) was that, in spite of the therapeutic benefits of the ENT procedure, the sinus sampling is not an appropriate microbiological approach for the diagnosis of the intracranial suppurative complication because of the contaminating nasal flora that can prevent a correct diagnosis and treatment. In the present survey, only the microbiological data coming from the intracranial sampling was considered.

The goal of the study provided by Hall and coworkers was to assess the possible relationship between COVID-19 infection and occurrence of intracranial empyema by retrospectively reviewing all intracranial empyema cases from 2016 to 2021 [22]. Overall, they found 16 cases, 6 of them belonging to the 2016–2019 period (incidence of 1.25 cases/year, 0.3% of all period-related surgical admissions) and 11 of them belonging to the 2020–2021 period (5.5 cases/year, 1.2% of all admissions). The increase of cases in the 2021–2021 period is evident, although the small number of patients does not allow a reliable statistical analysis. 4/11 cases were COVID-positive and showed some differences with the non-COVID children such as lower mean age (8.5 vs 11 years), presence of frontal and maxillary sinusitis with supratentorial empyema (100 vs 58%), need of ENT toilette (75% vs 0%), occurrence of sinus thrombosis (75% vs 25%). No differences in symptoms and outcome (full recovery) were detected. The associated literature review disclosed additional 5 cases of empyema with COVID-19 infection (2 adults and 3 children) showing full recovery in children but need of rehabilitation (one case) or death (one case) in adults. The authors concluded that intracranial empyema could be a potential non-respiratory sequela of COVID-19 and that the potential thrombogenic effects of COVID-19 increases the risk of sinus thrombosis. The present survey confirms the intensification of ICSO after the COVID era but disproves either a role of an active COVID-19 infection in causing these complications and a relationship with sinus thrombosis.

Finally, Angelo and coworkers performed a retrospective analysis on < 21-year-old patients with intracranial complications of sinusitis and otitis looking for possible differences in “epidemiology, severity, microbial causes, and management strategies” between pre-COVID-19 (2012–2020) and COVID-19 era (2021–2022) [21]. The authors collected 18 cases: 10 admitted in the 2012–2020 period (incidence of 1.25 cases/year) and 8 in the 2021–2022 period (4 cases/year). With the main limitation of the few number of patients, this study showed some important findings that are confirmed by the present survey on a larger sample: (1) no significant difference in sex (66.7% males), age, race, ethnicity and language distribution or insurance type between the two periods; (2) sinogenic (89%) or otogenic intracranial infection (11%) occurred with a rate of 2.64 ± 1.91 cases/10,000 acute respiratory infections in the 2015–2019 period vs 8.48/10,000 in 2021–2022 period (*p* = 0.03). This was the only statistically significant data in the study (apart from the prevalence of some germs and the need of seizure prophylaxis). (3) Headache (77.8%), fever (55.6%), and facial swelling (38.9%) were the most common presenting symptoms followed by vomiting (22.2%), periorbital swelling (22.2%), cough (16.7%), seizures (11.1%), and neck stiffness (5.6%). Epidural abscess was the most common complication (66.7%) followed by subdural empyema (33.3%) and brain abscess (5.6%). As far as the clinical and radiological presentation was concerned, no differences were detected between pre-COVID and COVID period in terms of type of symptoms, symptom onset, type of intracranial complication, previous care visit before the admission, preoperative imaging modality. (4) Similarly, no relevant differences were noticed between the two periods about the number and types of neurosurgical and ENT procedures. The authors acknowledged a threefold increase in cases of sinusitis- and otitis media–related intracranial infections compared with the baseline rate and the need of further studies to confirm this result in the US.

The goal of the present survey was to increase the number of cases and the number of involved centers trying to obtain representative results in Europe. Actually, the strength of this study is the high number of patients and their multicenter origin that allow to draw some conclusions on the epidemiology of sinogenic/otogenic intracranial complications and their sequelae in the pre-COVID, COVID, and post-COVID period. The main limitations are related to the retrospective analysis, the voluntary participation to the study (which may not reflect the real trend of infections throughout Europe), and the possible different management strategies among the different centers.

### Epidemiological considerations

ICSO are known to be rare but potentially severe complications, raising a great interest among the pediatric neurosurgical community [24, 25]. Their occurrence was variously described, being reported as estimated prevalence of 1/193,000 people [26], incidence of 3/million people/year [27], or admission rate of 2.74–4.38/million children/year [28]. Children and young adults are more commonly involved than young children or infants because of the pneumatization of the frontal sinuses that becomes complete only in older children (around 10 years of age) [29, 30]. The present survey confirms this data, showing an overall mean age of 10.17 years, which does not show significant changes along all the period considered (2017–2023) nor between the pre-COVID (2017–2019), the COVID (2020–2021), and the post-COVID era (2022–2023) (Table 2).

The most important result of the present study is the significant increase in frequency of the aforementioned complications after the pandemic (169 cases in the 2020–2023 period) compared with the previous period (85 cases in the 2017–2019 period) in spite of the higher incidence of predisposing risk factors/concurrent medical conditions (different from COVID) in the latter. In particular, the most relevant data was the statistically significant increase in the number of cases in the post-COVID era (129 cases in the 2022–2023 period, which was only 1.5-year long since the survey was closed in June 2023) compared with both the COVID (40 cases in the 2020–2021 period) and the pre-COVID era (85 cases in the 2017–2019 period). This overall trend was noticed in 60% of participating centers (Table 1). It is worth noting that these figures probably underestimate the real incidence of ICSO because the survey was focused on surgical patients. These results demonstrate that (1) the increase of ICSO correlates with the post-pandemic era. Indeed, differently from the study by CDC, where an increase was noticed in 2021 and a peak was reached in 2022 (comparable with a seasonal peak in 2016) followed by a decline to baseline [9], the present study shows a constant increase through 2022 and 2023 which is higher than the baseline registered during the 2017–2019 period. The main limitation of the CDC study, acknowledged by the authors themselves, is the use of an administrative database for their research. Moreover, the study was focused on streptococcus infections. On the other hand, the present study is corroborated by a multicenter, clinical analysis where different European countries (with their different ethnicities, weather, habits, types of bacterial infection, etc.) were involved. (2) During the “active” phase of the pandemic (2020–2021, COVID period), there was a reduction of cases which is easily explained by the historically low occurrence of acute respiratory infections during the lockdown period. Such a reduction was actually experienced also by Angelo et al. who had no cases of intracranial infections in 2020 (compared with 10 cases in the 2012–2019 period and 8 cases in the 2021–2022 period) [21]. (3) The post-COVID period (2022–2023) was the most involved one. The definition of these 18 months as “post-COVID period” is, of course, arbitrary but it corresponds quite homogeneously to the post-lockdown phase in Europe. This phase is clearly pointed as the most critical one since the number of cases collected in 2022 and, especially, in the first half of 2023 is higher compared with all the previous years (Fig. 1). A first explanation of these figures would come from a direct effect of COVID-19 infection. Indeed, the previous COVID-infection rate was significantly higher in the 2022–2023 period than in the 2020–2021 period and, obviously, in the 2017–2019 period (where COVID was absent). A possible limitation of this data is related to the lower number of COVID tests performed during the lockdown period and/or to the higher reliability of those available after the lockdown phase. On the other hand, the statistically higher incidence of other risk factors found in the pre-COVID period indirectly supports the role of COVID infection in the other two periods. However, it is worth noting that a previous COVID infection affected only 22% of cases in the 2022–2023 period; therefore, the direct effect of COVID infection can be considered as one possible but not the only/main risk factor for complicated sinusitis.

Two main hypotheses can be advocated to explain the direct role of COVID infection. The first one concerns the immunomodulatory effects of COVID-19. As for other viral infection, indeed, COVID-19 can cause a dysregulation of the innate and adaptative immune response [31–33]. In turn, such a dysregulation is able to favor a bacterial superinfection [8, 34]. On these grounds, it has been postulated that COVID-19 could act as some other viruses (e.g., influenza virus) in potentiating the bacterial virulence and the penetration through the respiratory epithelia by impairing the mucociliary function, depleting macrophages, downregulating antimicrobial peptides, increasing bacterial receptor exposure, and enhancing mucosal inflammation [35, 36]. This theory is supported by the evidence of two factors potentially favoring the bacterial penetration in adults: the inflammation of the respiratory tract mucosa and the circulation of COVID DNA and lipopolysaccharide in the plasma [37, 38]. Nevertheless, the role of COVID-19 in favoring the bacterial penetration in the central nervous system still remains unproved in children [21, 22]. The second hypothesis concerns the impairment of the respiratory microbiota. Once again, a certain evidence about the role of the quality of oropharyngeal colonization in enhancing the COVID-19 virulence has been found in adults [39] while the role of the respiratory tract microbiota composition in children remains speculative [40, 41]. Controversial remains also the role of COVID-19 in influencing the occurrence of sinusitis and otitis because, on the one hand, COVID infection can favor bacterial superinfection and immune dysregulation (thus favoring sinusitis/otitis) [42] while, on the other hand, lockdown, masking, and vaccination could have contributed to their reduction [43].

A second explanation comes from the indirect effects of the pandemic. In this instance, three main hypotheses can be proposed. The first one concerns the social distance and the mask wearing which allowed a significant decrease of the respiratory tract infections during the lockdown period, thus preventing also the children’s exposure to endemic pathogens and altering the composition of their normal bacterial flora [44–47]. Such an immunity debt would have been responsible of the decline of the immune response to infection thus explaining the increase of viral (e.g., respiratory syncytial virus) and bacterial infection detected worldwide in the post-lockdown period [44, 48–51]. Although this is still unproved and controversial, the present survey suggests that this immunity debt could have played a role in rising the risk of sinusitis and otitis and, so, their intracranial complications because most of the involved children did not show specific risk factors (including active or previous COVID infection). The second hypothesis is also based on the societal effects of the pandemic. In this instance, the difficult access to medical resources is advocated. Indeed, namely, during the lockdown period, several diseases with usually benign course (like sinusitis or otitis) were considered as not priority so that their delayed treatment could have increased the risk of their complications [52, 53]. One of the strength points of this survey is to have considered the post-COVID period where the access to the medical resources was normalized. On this base, the role of a poor access to the standard care should be considered as negligible in increasing ICSO. The third hypothesis concerns the potential side effects of vaccination. To date, however, there are only a few and isolated case reports speculating on a possible correlation between COVID vaccination and neurological side effects [54, 55] or occurrence of venous sinus thrombosis [56] or increased risk of sinusitis/otitis [57]. Large trials, indeed, have demonstrated the safety of COVID vaccinations on these potential complications [58]. A possible role of vaccination, therefore, should be considered for future, specific studies [59].

The present analysis showed a prevalence of the male sex (overall M/F ratio: 1.9) without significant fluctuations over the years nor among the 3 main examined periods, though a progressive increase of the M/F ratio was noticed (1.6 vs 1.8 vs 2.14) (Table 2). A male preponderance is demonstrated by studies specifically investigating the sex differences in rhinosinusitis [60]. Actually, males are found to present a complex anatomy (concha bullosa, supraorbital ethmoid cell) that significantly favors sinusitis [61, 62]. As a result, ICSO are more frequent in males with a M/F ratio of up to 3 [30, 63]. According to the present survey, therefore, the pandemic did not influence such a male predominance.

Similarly, the pathogen spectrum remained relatively stable over the time, taking into account the obvious variations in the identified bacteria depending on the different countries involved in the study (Table 5). Actually, *Streptococcus* bacteria (namely, *S. intermedius* followed by *S. anginosus*, *S. constellatus*, *S. pyogenes*, and *S. pneumoniae*) were largely predominant in all the different periods as found also in other similar studies [9, 23, 63]. Inversely, Angelo et al. found a difference between the microorganisms identified in the pre-COVID (mainly *Corynebacterium*, *Staphylococcus epidermidis*, and viridans streptococci) and in the COVID era (mainly *Streptococcus constellatus*/*Streptococcus anginosus*/*Streptococcus intermedius*, and *Parvimonas micra*) though with the significant limitation of the small number of cases [21]. On the other hand, a concordance among the different studies exists about the occurrence of polymicrobial cultures and negative intracranial cultures (19% in the Kameda-Smith series, 31% in the present survey) [9, 21, 23, 63, 64].

**Fig. 3:**
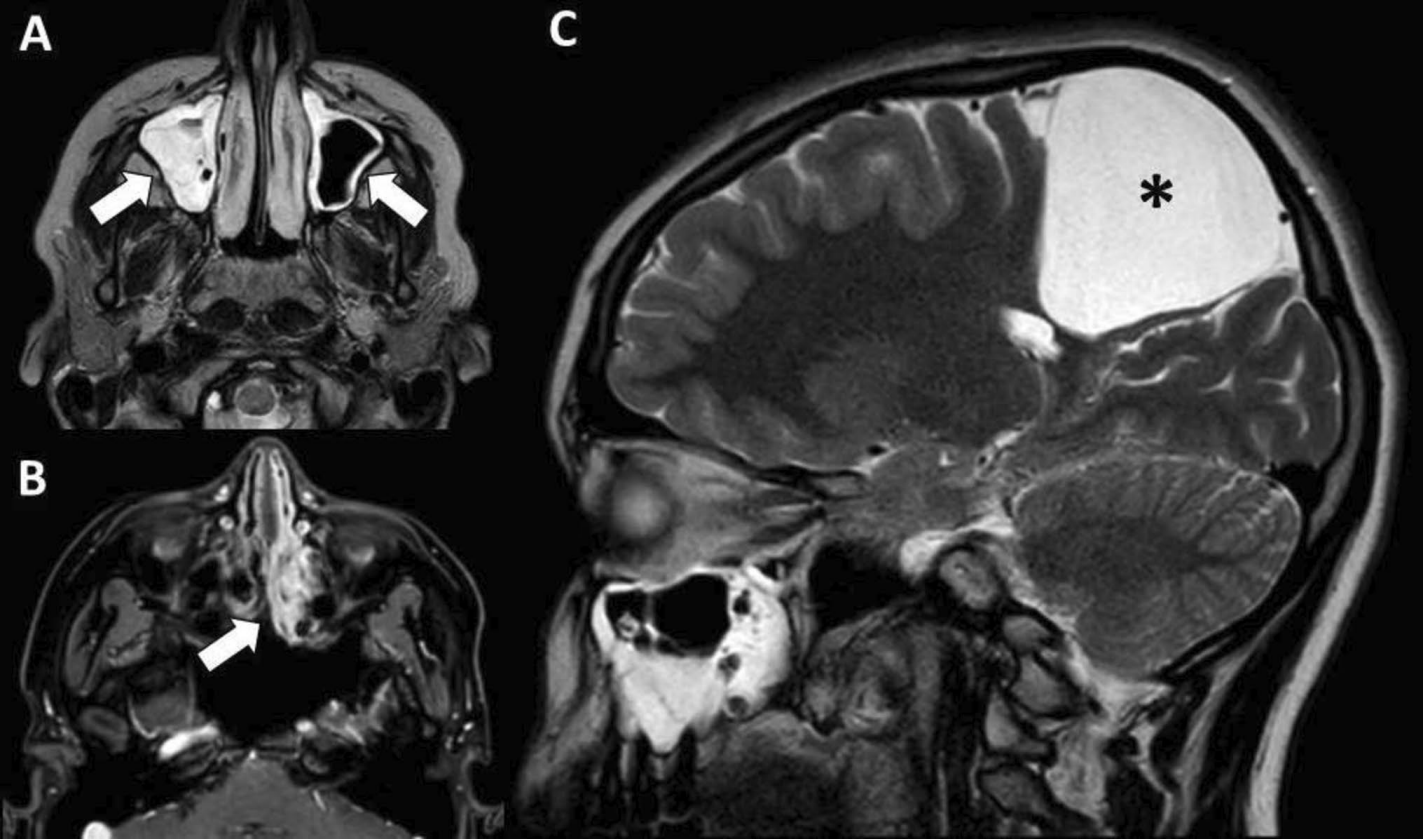
Incidental radiological findings of sinusitis involving the maxillary sinuses and ethmoid cells (**A** and **B**, arrows) at routine follow-up MR for arachnoid cyst (**C**, asterisk)

This epidemiological excursus shows that the risk of ICSO was increased after the pandemic because of the increased occurrence of sinusitis and otitis probably resulting from indirect more than direct effects of COVID-19. On these grounds, this condition will deserve a special attention in the future, though the incidental findings of sinusitis (Fig. 3) or opacification of middle ear space are normal with the increasing number of radiological examinations in the population and the incidence rates of upper respiratory tract infections and otitis media with effusion especially in pre-school children.

### Clinical considerations

Apart from the singenic and/or otogenic origin, which covers the majority of cases (40–80%), intracranial empyema and brain abscess can result also from cranial surgery, head injury, or hematogeneous spread [65, 66]. The complications of neurosurgical procedures, head trauma, and pulmonary/other infections were excluded in the present survey because they are not related with the pandemic. ICSO result from a septic retrograde thrombophlebitis originating inside the infected cavity [23]. The bacteria would penetrate the intracranial space via the diploic veins connecting the sinus/ear mucosa and the intracranial venous system [25]. Such a penetration usually results in a subdural empyema. When a focal osteitis is present, an extradural empyema can occur (that, in turn, can extend into the subdural space) while, in case of hematogeneous spreading inside the cranial space, multiple areas can be involved (including the brain parenchyma). In general, the present survey shows that paranasal sinuses were the most common source of intracranial infection (71%) followed by middle ear or other locations (29%). Extradural (38%) and subdural space (37%) were equally distributed and followed by brain abscess (17%) and other complications (10%). This does not differ significantly from what was found in similar studies where, sometimes, the extradural collection was prevalent on the subdural one and vice versa [21–23]. No significant variation in the distribution of the complications was found across the different years and the 3 periods, thus suggesting that the increase of the number of cases does not affect the type of complications. Moreover, this study demonstrated a significant proportion of cases with multiple locations (22%) (Table 4). This new data justifies the significant morbidity previously acknowledged by other authors [28, 67]. Actually, the present study revealed a relevant rate of patients presenting onset symptoms different from fever (84%), with high rate of focal neurological deficits (31%), seizures (15%), or coma (12%). Also, the overall mortality rate was not trivial (2.7%), thus confirming the potential severity of ICSO. The homogeneous distribution of neurological presenting symptoms and signs during the different years and periods (Table 4) together with the low number of COVID infection (Table 3) allows to ascribe the occurrence of these symptoms to the empyema/abscess rather than to a central nervous system involvement by the COVID infection. Actually, although COVID-19 has been found to be responsible of meningitis and several types of encephalopathies [68–72], this event can be considered as marginal in the present series.

Among the intracranial complication, the venous sinus thrombosis deserves a special mention because a specific, possible correlation with the COVID infection has been postulated. Venous sinus thrombosis, indeed, is a well-known complication of intracranial empyema, occurring in about 10% of cases, usually going along with worse prognosis [73]. However, several studies showed that COVID infection itself is associated with a risk of venous sinus thrombosis, probably because of the hypercoagulability induced by COVID-19, resulting in a significant mortality rate (about 36%) [74, 75]. Based on their data of 75% of children with venous sinus thrombosis among those with intracranial empyema and recent COVID infection (3 out of 4 cases) versus 25% of children without COVID infection (3 out of 12 cases), Hall et al. postulated a concurrent role of COVID infection in inducing sinus venous thrombosis in this subset of patents [22]. The present survey does not confirm this data in a large number of cases. Actually, despite venous sinus thrombosis occurred in a significant proportion of cases in the whole series (17%), it was not correlated with the presence or a previous COVID-19 infection, being distributed without statistically significant variations over the time, including the pre-COVID period (Table 4).

On the other hand, this survey confirms the management strategies usually adopted to face ICSO, without significant variations in the 3 periods considered (Tables 5 and 6). Indeed, the standard care, in these instances, is represented by neurosurgical evacuation of the intracranial, infected collection plus systemic antibiotic treatment (generally for 4 to 8 weeks) [21, 76, 77]. The neurosurgical procedure is necessary to improve the clinical picture as well as to obtain specimens for microbiological cultures. Subsequently, it was performed in the great majority of cases in the present series, without variations over the time, only 5% of cases being treated successfully with empiric antibiotics alone. It is worth noting that, in the present series, the evacuation of the intracranial collection (namely, epidural empyema) was sometimes obtained through an ENT toilette of the infected cavity. Actually, the number of ENT procedures was as high as 62% in the whole series (largely stable rate over the time), thus confirming their role not only in cleaning the infected paranasal sinuses/middle ear but also in evacuating extradural empyema, reducing the risk of brain abscess and the risk of revision surgery [78, 79]. This observation justifies the high incidence of ENT procedures (90%) in some series [21]. Overall and without considering additional procedures (e.g., decompressing craniectomy, external ventriculostomy, neuroendoscopic lavage), a neurosurgical operation purely devoted to the evacuation of the intracranial complication was realized in 71% of cases. Together with the number of ENT operations, this resulted in a high rate of multiple operation with a 13% overall rate of more than 3 surgical procedures (the relatively low number of > 3 operations in 2023 can be explained by the short follow-up in this year). This data gives reason of the complex management of these complications. A further proof of such a complex management is represented by the high number of performed craniotomies (48% in the whole series) compared with simple burr-hole evacuations (23%). This finding, once again essentially unchanged over the time, is partially comparable with other studies where craniotomy approaches were used in up to 70–80% of cases compared with burr holes (7.5–39%) [21, 23, 80]. Of course, these figures in part reflect also the different management policies adopted in the different centers.

Similarly, some details on the medical treatments confirm the complex management of ICSO. First of all is the duration of the antibiotic treatment. In the whole series, it was meanly 5.8-week long, as quite analogously reported by other studies [21, 23]. Moreover, a not negligible rate of children needed a more than 10 weeks of treatment (14%), once again the relatively low rate in 2023 (7%) being attributable to the shorter follow-up (some patients were still under treatment when the survey was closed). As showed by other authors, due to the long duration of medical therapy, often patients are discharged still with a combination of intravenous (mainly ceftriaxone or meropenem) and oral antibiotics (usually metronidazole) [21]. A second clue of the complexity of the management is the frequent use of steroids and/or anti-inflammatory drugs. This accounted for 56% of cases in the whole series, with no relevant variations over the time. These drugs are mainly required to manage symptoms like fever, meningitis signs, or headache.

Some final, clinical remarks can be given about the outcome of ICSO. As known, in spite of good survival rates (usually > 90%), intracranial empyema/abscess can leave permanent neurological deficits in up to 50% of cases in some series [64, 81, 82]. These figures are here confirmed since, despite a complete recovery in 85% of cases, the remaining 15% of children show permanent neurological sequelae like hemiparesis, seizures, spasticity or cranial nerve palsy (Table 7). This is not surprising given the relatively high rate of subdural (37%) or brain involvement (17%), multiple intracranial infections (22%), and venous sinus thrombosis (17%) (Table 4). Under a clinic-epidemiological point of view, once again, the course of the complications was not affected by the period where they occurred. Comparable data is provided by Angelo et al. who found 2 cases each (20%) with permanent neurological deficits and seizures in the pre-COVID period and 12.5% (1 case) and 25% (2 cases), respectively, in the COVID period [21].

Similar considerations can be done about the mortality. Actually, apart from some small series, where no mortality occurred [21, 80], fatal events are detected in larger series, ranging from 6 to 16%, especially if older or conservatively managed patients are included [83–85]. The lower mortality rate in this survey (2.7%) probably depends, at least in part, on the purely pediatric series and the high number of surgeries performed (95% of cases). However, such a figure is not negligible as it underlies the potential severity of ICSO, in particular when there is a brain involvement. Indeed, the main causes of death were post-meningitis encephalopathy, brain edema, and central dysregulation. The persistence of some radiological sequelae (overall, 16%) after a mean 12.4-month follow-up, like meningeal impregnation or gliosis, is part of the normal evolution of this kind of infection that does not prevent a full recovery [21, 83]. In the present series, indeed, only two cases of recurrent infection occurred (0.7%, both subdural empyema).

## Conclusions

This survey shows that ICSO are clinically relevant and, in particular, they have been increasing in the last years. Namely, a significant growth of the number of cases is detected in the post-COVID period, although not homogeneously in all participating centers. Therefore, a partial role of seasonal and/or environmental fluctuations cannot be definitely excluded but the general trend is clearly in favor of a raised impact of these complications on the daily clinical practice. The most important aspect of this survey, indeed, is to have showed a possible epidemiological change in the problem (sinusitis/otitis) and to alert about the possible sequelae (intracranial empyema and abscess) and the need of an appropriate follow-up of children with sinusitis/otitis. This study cannot rule out a direct role of COVID infection in increasing the number of sinusitis and their complications in a minority of cases. On the other hand, it seems reasonable to hypothesize a more substantial role of the indirect effects of the pandemic in favoring these events. The continuation of a longer survey and/or data coming from other surveys and/or prospective studies will allow to solve this matter. Finally, the pandemic did not affect the management strategies of these intracranial complications nor their prognosis.

### Supplementary Information

Below is the link to the electronic supplementary material.

Supplementary file1 (DOCX 22.9 KB)

## Data Availability

The data that support the findings of this study are not openly available due to reasons of sensitivity and are available from the corresponding author upon reasonable request.

## References

[1] Capozza MA, Triarico S, Attinà G (2021). Managing children with brain tumors during the COVID-19 era: don’t stop the care!. Comput Struct Biotechnol J.

[2] Lee MH, Jang S-R, Lee T-K (2023). The direction of neurosurgery to overcome the living with COVID-19 era : the possibility of telemedicine in neurosurgery. J Korean Neurosurg Soc.

[3] Dong J, Wang S, Xie H (2023). COVID-19 hospitalization increases the risk of developing glioblastoma: a bidirectional Mendelian-randomization study. Front Oncol.

[4] Karamani L, McLean AL, Kamp MA, Mayer TE, Müller W, Dinc N, Senft C (2023). Tumor size, treatment patterns, and survival in neuro-oncology patients before and during the COVID-19 pandemic. Neurosurg Rev.

[5] Leiphart TJ, Leiphart J (2023). The effect of the COVID-19 pandemic and lockdown on operative traumatic brain injury in Northern Virginia. Cureus.

[6] Troy BM, Fraser Doh K, Linden AF, Xiang Y, Gillespie S, Agarwal M (2023). Changes in pediatric injuries sustained while engaged in activities where helmet usage is recommended during the COVID-19 pandemic. Inj Epidemiol.

[7] Arteaga AA, Tran J, Frey H, Lewis AF (2022). Rapidly progressive complicated acute bacterial sinusitis in the setting of severe pediatric SARS-CoV-2 infection. Ann Otol Rhinol Laryngol.

[8] Turbin RE, Wawrzusin PJ, Sakla NM, Traba CM, Wong KG, Mirani N, Eloy JA, Nimchinsky EA (2020). Orbital cellulitis, sinusitis and intracranial abnormalities in two adolescents with COVID-19. Orbit.

[9] Accorsi EK, Chochua S, Moline HL (2022). Pediatric brain abscesses, epidural empyemas, and subdural empyemas associated with Streptococcus species - United States, January 2016-August 2022. Morb Mortal Wkly Rep.

[10] Khuon D, Ogrin S, Engels J, Aldrich A, Olivero RM (2022). Notes from the field: increase in pediatric intracranial infections during the COVID-19 pandemic - eight pediatric hospitals, United States, March 2020-March 2022. Morb Mortal Wkly Rep.

[11] Parisi GF, Diaferio L, Brindisi G (2021). Cross-sectional survey on long term sequelae of pediatric COVID-19 among Italian pediatricians. Children (Basel).

[12] Sheikh A, Capello C, AlMubarak Z, Dzioba A, You P, Nashid N, Barton M, Husein M, Strychowsky JE, Graham ME (2023). Changes in operative otolaryngology infections related to the COVID19 pandemic: a retrospective cohort study. Int J Pediatr Otorhinolaryngol.

[13] Sutton SR, Taniguchi AN, Nguyen SA, Soler ZM, Schlosser RJ (2023). Direct impact of the COVID-19 pandemic on rhinology practice. Int Forum Allergy Rhinol.

[14] Blumfield E, Misra M (2011). Pott’s puffy tumor, intracranial, and orbital complications as the initial presentation of sinusitis in healthy adolescents, a case series. Emerg Radiol.

[15] Wald ER, Applegate KE, Bordley C (2013). Clinical practice guideline for the diagnosis and management of acute bacterial sinusitis in children aged 1 to 18 years. Pediatrics.

[16] Arcalas C-JE, Reich DA, Blair SA, Paradise Black NM (2023). Acute bacterial sinusitis with epidural and subdural involvement. Cureus.

[17] Milinis K, Thiagarajan J, Leong S (2023). Review of management practices of sinogenic intracranial abscesses in children. J Laryngol Otol.

[18] Nicoli TK, Oinas M, Niemelä M, Mäkitie AA, Atula T (2016). Intracranial suppurative complications of sinusitis. Scand J Surg.

[19] Hong CS, Prust ML, Manes RP, Rimmer RA, Omay SB (2023). Subdural empyema secondary to pansinusitis after coronavirus disease 2019 infection in an immunocompetent patient: illustrative case. J Neurosurg Case Lessons.

[20] Ljubimov VA, Babadjouni R, Ha J, Krutikova VO, Koempel JA, Chu J, Chiarelli PA (2022). Adolescent subdural empyema in setting of COVID-19 infection: illustrative case. J Neurosurg Case Lessons.

[21] Angelo SJ, Anderson MG, Sutter PA, Halloran PJ, Kavanagh KR, Paro MR, Martin JE, Bookland MJ, Michelow IC, Hersh DS (2023). Changes in the epidemiology of pediatric sinogenic and otogenic intracranial infections during the COVID-19 pandemic: a single-institution study. J Neurosurg Pediatr.

[22] Hall B, Duddy JC, Apostolopoulou K (2023). Intracranial empyemas in the COVID-19 era: a new phenomenon? A paediatric case series and review of the literature. Pediatr Neurosurg.

[23] Kameda-Smith MM, Mendoza M, Brown L-A (2023). Comparison of endoscopic sinus sampling versus intracranial sampling for microbiological diagnosis of intracranial infection in children: a case series and literature review. Childs Nerv Syst.

[24] Gilchrist JJ, Hoy T, Bijker EM, Lees EA, Wilkins L, Oliver M, Kelly DF, Paulus SC, Calisto A (2023). Intracranial empyema in children: a single-center retrospective case series. Pediatr Infect Dis J.

[25] Hakim HE, Malik AC, Aronyk K, Ledi E, Bhargava R (2006). The prevalence of intracranial complications in pediatric frontal sinusitis. Int J Pediatr Otorhinolaryngol.

[26] Nathoo N, Nadvi SS, van Dellen JR, Gouws E (1999). Intracranial subdural empyemas in the era of computed tomography: a review of 699 cases. Neurosurgery.

[27] Fokkens WJ, Lund VJ, Hopkins C (2020). European position paper on rhinosinusitis and nasal polyps 2020. Rhinology.

[28] Piatt JH (2011). Intracranial suppuration complicating sinusitis among children: an epidemiological and clinical study. J Neurosurg Pediatr.

[29] Adibelli ZH, Songu M, Adibelli H (2011). Paranasal sinus development in children: a magnetic resonance imaging analysis. Am J Rhinol Allergy.

[30] De Bonis P, Anile C, Pompucci A, Labonia M, Lucantoni C, Mangiola A (2009). Cranial and spinal subdural empyema. Br J Neurosurg.

[31] Ingraham NE, Lotfi-Emran S, Thielen BK, Techar K, Morris RS, Holtan SG, Dudley RA, Tignanelli CJ (2020). Immunomodulation in COVID-19. Lancet Respir Med.

[32] Rowntree LC, Nguyen THO, Kedzierski L (2022). SARS-CoV-2-specific T cell memory with common TCRαβ motifs is established in unvaccinated children who seroconvert after infection. Immunity.

[33] Yoshida M, Worlock KB, Huang N (2022). Local and systemic responses to SARS-CoV-2 infection in children and adults. Nature.

[34] Carvalho VA, de Oliveira Vergínio VE, Brito GC, Pereira-Stabile CL, Stabile GAV (2021). Coronavirus disease 2019 as a possible cause of severe orbital cellulitis. J Craniofac Surg.

[35] Choe YJ, Park S, Michelow IC (2020). Co-seasonality and co-detection of respiratory viruses and bacteraemia in children: a retrospective analysis. Clin Microbiol Infect.

[36] McCullers JA (2014). The co-pathogenesis of influenza viruses with bacteria in the lung. Nat Rev Microbiol.

[37] Arunachalam PS, Wimmers F, Mok CKP (2020). Systems biological assessment of immunity to mild versus severe COVID-19 infection in humans. Science.

[38] Baker JR, Mahdi M, Nicolau DV, Ramakrishnan S, Barnes PJ, Simpson JL, Cass SP, Russell REK, Donnelly LE, Bafadhel M (2022). Early Th2 inflammation in the upper respiratory mucosa as a predictor of severe COVID-19 and modulation by early treatment with inhaled corticosteroids: a mechanistic analysis. Lancet Respir Med.

[39] Hernández-Terán A, Mejía-Nepomuceno F, Herrera MT (2021). Dysbiosis and structural disruption of the respiratory microbiota in COVID-19 patients with severe and fatal outcomes. Sci Rep.

[40] Hurst JH, McCumber AW, Aquino JN (2022). Age-related changes in the nasopharyngeal microbiome are associated with severe acute respiratory syndrome coronavirus 2 (SARS-CoV-2) infection and symptoms among children, adolescents, and young adults. Clin Infect Dis.

[41] Lee SE, Ghodke AN, Stepp WH, Kong KA, Chaskes M, Quinsey CS, Ebert CS, Thorp BD, Senior BA, Kimple AJ (2023). Sinonasal complications of severe acute respiratory syndrome coronavirus-2: a single center case series. Laryngoscope Investig Otolaryngol.

[42] Abi Zeid Daou C, Yammine Y, Daou A-M, Feghali PAR, Najjar W, Barazi R (2023). Incidence of pediatric tonsillitis, otitis and upper respiratory infectious entities in the pre and post COVID-19 quarantine eras. Acta Otolaryngol.

[43] Favoretto MH, Mitre EI, Vianna MF, Lazarini PR (2022). The impact of COVID-19 pandemic on acute otitis media among the pediatric population. Int J Pediatr Otorhinolaryngol.

[44] Cohen R, Ashman M, Taha M-K, Varon E, Angoulvant F, Levy C, Rybak A, Ouldali N, Guiso N, Grimprel E (2021). Pediatric Infectious Disease Group (GPIP) position paper on the immune debt of the COVID-19 pandemic in childhood, how can we fill the immunity gap?. Infect Dis Now.

[45] Eyre TA, Peters L, Andersson MI, Peniket A, Eyre DW (2021). Reduction in incidence of non-COVID-19 respiratory virus infection amongst haematology inpatients following UK social distancing measures. Br J Haematol.

[46] Rocafort M, Henares D, Brotons P (2022). Impact of COVID-19 lockdown on the nasopharyngeal microbiota of children and adults self-confined at home. Viruses.

[47] Tanislav C, Kostev K (2022). Fewer non-COVID-19 respiratory tract infections and gastrointestinal infections during the COVID-19 pandemic. J Med Virol.

[48] Billard M-N, van de Ven PM, Baraldi B, Kragten-Tabatabaie L, Bont LJ, Wildenbeest JG (2022). International changes in respiratory syncytial virus (RSV) epidemiology during the COVID-19 pandemic: association with school closures. Influenza Other Respir Viruses.

[49] Foley DA, Phuong LK, Peplinski J (2022). Examining the interseasonal resurgence of respiratory syncytial virus in Western Australia. Arch Dis Child.

[50] Hodjat P, Christensen PA, Subedi S, Bernard DW, Olsen RJ, Long SW (2021). The reemergence of seasonal respiratory viruses in Houston, Texas, after relaxing COVID-19 restrictions. Microbiol Spectr.

[51] Maison N, Peck A, Illi S, Meyer-Buehn M, von Mutius E, Hübner J, von Both U (2022). The rising of old foes: impact of lockdown periods on “non-SARS-CoV-2” viral respiratory and gastrointestinal infections. Infection.

[52] Radhakrishnan L, Carey K, Hartnett KP (2022). Pediatric emergency department visits before and during the COVID-19 pandemic - United States, January 2019-January 2022. Morb Mortal Wkly Rep.

[53] Shukla P, Lee M, Whitman SA, Pine KH (2022). Delay of routine health care during the COVID-19 pandemic: a theoretical model of individuals’ risk assessment and decision making. Soc Sci Med.

[54] Finsterer J (2022). A case report: long post-COVID vaccination syndrome during the eleven months after the third Moderna dose. Cureus.

[55] Kalita IR, Singh HV, Sharma S (2023). Acute abducens nerve palsy with acute disseminated encephalomyelitis-like presentation following COVID-19 vaccination. Indian J Ophthalmol.

[56] Alunni V, Bernardi C, Chevalier N, Cabusat C, Quatrehomme G, Torrents J, Biglia E, Gaillard Y, Drici M-D (2023). Postmortem PF4 antibodies confirm a rare case of thrombosis thrombocytopenia syndrome associated with ChAdOx1 nCoV-19 anti-COVID vaccination. Int J Legal Med.

[57] Cline L, Nguyen HT, Olenik A (2022). Cerebral venous sinus thrombosis following COVID-19 and otogenic infection: a diagnostic and therapeutic dilemma followed by mRNA COVID-19 vaccination. Perm J.

[58] Ahsanuddin S, Jin R, Dhanda AK, Georges K, Baredes S, Eloy JA, Fang CH (2023). Otolaryngologic side effects after COVID-19 vaccination. Laryngoscope.

[59] Beghi E, Ivashynka A, Logroscino G, de Oliveira FF, Fleisher JE, Dumitrascu OM, Patel R, Savica R, Kim YJ (2023). Pitfalls and biases in neuroepidemiological studies of COVID-19 and the nervous system: a critical appraisal of the current evidence and future directions. J Neurol.

[60] Asokan A, Mace JC, Rice JD, Smith TL, Soler ZM, Ramakrishnan VR (2023). Sex differences in presentation and surgical outcomes from a prospective multicenter chronic rhinosinusitis study. Otolaryngol Head Neck Surg.

[61] Howser LA, Jones AJ, Sreenath SB, Ting JY, Illing EA (2023) Frontal sinus anatomy variations in race and sex using the international frontal sinus anatomy classification. Ear Nose Throat J 1455613231185701. 10.1177/01455613231185701 10.1177/01455613231185701 37470260

[62] Velasquez N, Strober W, Shaffer A, Stapleton A (2021). Clinical and radiologic characterization of frontal sinusitis in the pediatric population. Ann Otol Rhinol Laryngol.

[63] Osborn MK, Steinberg JP (2007). Subdural empyema and other suppurative complications of paranasal sinusitis. Lancet Infect Dis.

[64] French H, Schaefer N, Keijzers G, Barison D, Olson S (2014). Intracranial subdural empyema: a 10-year case series. Ochsner J.

[65] Bonfield CM, Sharma J, Dobson S (2015). Pediatric intracranial abscesses. J Infect.

[66] Suthar R, Sankhyan N (2019). Bacterial infections of the central nervous system. Indian J Pediatr.

[67] Hutton D, Kameda-Smith M, Afshari FT (2023). Intracranial invasive group A streptococcus: a neurosurgical emergency in children. J Neurosurg Pediatr.

[68] Bernard-Valnet R, Perriot S, Canales M (2021). Encephalopathies associated with severe COVID-19 present neurovascular unit alterations without evidence for strong neuroinflammation. Neurol Neuroimmunol Neuroinflamm.

[69] Boldrini M, Canoll PD, Klein RS (2021). How COVID-19 affects the brain. JAMA Psychiat.

[70] Duong L, Xu P, Liu A (2020). Meningoencephalitis without respiratory failure in a young female patient with COVID-19 infection in Downtown Los Angeles, early April 2020. Brain Behav Immun.

[71] Kantonen J, Mahzabin S, Mäyränpää MI (2020). Neuropathologic features of four autopsied COVID-19 patients. Brain Pathol.

[72] Naz S, Hanif M, Haider MA, Ali MJ, Ahmed MU, Saleem S (2020). Meningitis as an initial presentation of COVID-19: a case report. Front Public Health.

[73] Konar S, Gohil D, Shukla D, Sadashiva N, Uppar A, Bhat DI, Srinivas D, Arimappamagan A, Devi BI (2019). Predictors of outcome of subdural empyema in children. Neurosurg Focus.

[74] Abouhashem S, Eldawoody H, Taha MM (2021). Cerebral venous sinus thrombosis in patients with COVID-19 infection. Interdiscip Neurosurg.

[75] Nwajei F, Anand P, Abdalkader M (2020). Cerebral venous sinus thromboses in patients with SARS-CoV-2 infection: three cases and a review of the literature. J Stroke Cerebrovasc Dis.

[76] Muzumdar D, Jhawar S, Goel A (2011). Brain abscess: an overview. Int J Surg.

[77] Patel NA, Garber D, Hu S, Kamat A (2016). Systematic review and case report: intracranial complications of pediatric sinusitis. Int J Pediatr Otorhinolaryngol.

[78] McNeil JC, Dunn JJ, Kaplan SL, Vallejo JG (2020). Complications of otitis media and sinusitis caused by Streptococcus anginosus group organisms in children. Pediatr Infect Dis J.

[79] Tandon S, Beasley N, Swift AC (2009). Changing trends in intracranial abscesses secondary to ear and sinus disease. J Laryngol Otol.

[80] Güçlü DG, Dolen D, Dolaş İ (2023). Surgical management of interhemispheric subdural empyemas: review of the literature and report of 12 cases. Ulus Travma Acil Cerrahi Derg.

[81] Dill SR, Cobbs CG, McDonald CK (1995). Subdural empyema: analysis of 32 cases and review. Clin Infect Dis.

[82] Fernández-de Thomas RJ, De Jesus O (2023) Subdural Empyema. [Updated 2023 Aug 23]. In: StatPearls [Internet]. Treasure Island (FL): StatPearls Publishing; 2024 Jan-. Available from: https://www.ncbi.nlm.nih.gov/books/NBK557829/32491761

[83] Banerjee AD, Pandey P, Devi BI, Sampath S, Chandramouli BA (2009). Pediatric supratentorial subdural empyemas: a retrospective analysis of 65 cases. Pediatr Neurosurg.

[84] Jolayemi EO, Bankole OB, Ojo OA (2022). Contemporary management of intracranial subdural empyema: an institutional experience. J West Afr Coll Surg.

[85] Lundy P, Kaufman C, Garcia D, Partington MD, Grabb PA (2019). Intracranial subdural empyemas and epidural abscesses in children. J Neurosurg Pediatr.

