# Supplementary material for: Intracranial complications of sinogenic and otogenic infections in children: an ESPN survey on their occurrence in the pre-COVID and post-COVID era
Source: Childs Nerv Syst. 2024 Mar 8;40(4):1221–37. doi: 10.1007/s00381-024-06332-9 (PMC10973035; doi:10.1007/s00381-024-06332-9)
Supplement: Supplementary file 1 — Supplementary file1 (DOCX 22.9 KB) [file 381_2024_6332_MOESM1_ESM.docx]

Intracranial complications of infections of paranasal sinuses

An ESPN survey on the incidence of this complication in the pre-COVID and post-COVID era (from 2017 to 2023)

**Email***

**Sending doctor/Author to be included in the study***

**Institution***

**Year of the event***

**Patient identification code**  *(initials of name and surname + date of birth + first letter of the Institution)**

**Age of the patient** *(years)**

**Gender***

M

F

**Immunocompromised?***

yes

no

**Other favoring disease?***

yes

no

*if yes, please specify*

**COVID infection?***

previous

concurrent

no

**Signs and symptoms***

none (only radiological diagnosis)

fever

seizures

neurological deficit

comatous state

other

*if other, please specify*

**Primary site of infection***

paranasal sinuses

middle ear

mastoid

other

*if other, please specify*

**Type of intracranial complication***

epidural empyema

subdural empyema

brain abscess

cerebritis

dural impregnation

venous sinus thrombosis

other

*if other, please specify*

**Responsible germ**

yes

no

*if yes, please specify*

**Surgical treatment***

none

burhole evacuation

craniotomy

ENT toilette

other

*if other, please specify*

***If surgery, number of neurosurgical procedures***

**Medical treatment***

none

steroids

antinflammatory drugs

antibiotics

**Duration of medical treatment***(weeks)**

**Clinical outcome: complete recovery?***

yes

no

*if no, please specify*

**Radiological outcome: complete resolution?***

yes

no

*if no, please specify*

**Follow-up (months)***

**: compulsory fields*
